# Outcomes of Diabetic Ketoacidosis in Adults With End-Stage Kidney Disease: Retrospective Study Based on a National Database

**DOI:** 10.7759/cureus.24782

**Published:** 2022-05-06

**Authors:** Karthik Gangu, Sanket D Basida, Anitha Vijayan, Sindhu Avula, Aniesh Bobba

**Affiliations:** 1 Internal Medicine, University of Missouri, Columbia, USA; 2 Medicine, Pandit Deendayal Upadhyay Medical College, Rajkot, IND; 3 Nephrology, Washington University School of Medicine in St. Louis, St. Louis, USA; 4 Cardiovascular Medicine, University of Kansas Medical Center, Kansas City, USA; 5 Internal Medicine, John H. Stroger, Jr. Hospital of Cook County, Chicago, USA

**Keywords:** orotracheal intubation, in-hospital mortality, diabetic ketoacidosis, end stage kidney disease, diabetes mellitus

## Abstract

Introduction: The prevalence of diabetes mellitus (DM) in the United States has steadily increased over the past few decades. End-stage kidney disease (ESKD) and diabetic ketoacidosis (DKA) are among the most common chronic and acute complications of DM. Guidance on the management of DKA in ESKD is limited by lack of evidence. We investigated the in-hospital outcomes of patients hospitalized for DKA with underlying ESKD.

Methods: We carried out a retrospective cohort study and utilized the National Inpatient Sample (NIS) database from 2016 to 2018. International Classification of Diseases, Tenth Revision, Clinical Modification (ICD-10 CM) codes were used to identify adults (>18 yrs) diagnosed with DM and ESKD. We compared patients with DKA and ESKD to patients who had DKA with preserved renal function. The primary outcomes were rates of in-hospital mortality and mechanical ventilation.

Results:Out of 538,135 patients, 18,685 (3.74%) represented DKA patients with ESKD, and 519,450 (96.53%) represented DKA patients with preserved renal function. DKA with concomitant ESKD was more prevalent in a relatively older population (age>30 yrs) with female predominance (52.4%) (p<0.001). The mean age of males and females in the ESKD group was 46.2 (SD 12.7) and 43.7 (SD 13.6) years respectively. African American race and low socioeconomic status had a higher burden of ESKD. In-hospital mortality rate (adjusted OR= 1.12, p=0.56) and need for mechanical ventilation (adjusted OR= 1.11, p=0.25) did not differ significantly in the two groups but adjusted mean total hospitalization charge ($14,882) and mean length of stay (0.87) at the hospital were significantly higher in patients with DKA and ESRD than in those with preserved renal function.

Conclusion: DKA is associated with short-term morbidity, increased length of stay, and cost of hospitalization. There is a dearth of evidence-based guidance regarding DKA management in CKD and ESRD. Further studies looking into measures in the management of DKA in ESRD will help develop guidelines in management, decreasing morbidity, and cost of hospitalization.

## Introduction

Diabetes mellitus (DM) is one of the fastest-growing epidemics of the 21st century and the seventh leading cause of death in the United States (US). According to the Centers for Disease Control and Prevention (CDC), as of 2017, 34.2 million people have diabetes (10.5% of the US population) [1]. This number will rise to 62.8 million by 2045, as per the International Diabetes Federation [2].

DM affects various body systems and causes various micro and macro-vascular complications [3]. Chronic kidney disease (CKD) is an extremely common chronic complication of DM [4]. Diabetes is the leading cause of CKD and end-stage kidney disease (ESKD), with approximately 40-50% of patients on chronic dialysis having an underlying diagnosis of DM [5]. There are over 720,000 patients with ESKD in the US, and the number is still increasing [1]. The mortality rate in this group of patients remains high, with an average death rate of 166 per 1000 patients/year [1]. 

Diabetic ketoacidosis (DKA) is one of the most acute complications related to diabetes [4]. Treatment of DKA with ESKD or CKD has been associated with an increased risk of hypoglycemia and volume overload [6,7]. If not treated promptly, patients can develop severe metabolic acidosis and other complications [6]. Data on appropriate management of DKA in the ESKD population are limited.

Our study evaluated the outcomes in the patients hospitalized for DKA and the burden on healthcare and resource utilization in patients with ESKD using the largest nationwide inpatient database.

## Materials and methods

Data source

We utilized the National Inpatient Sample (NIS) from Agency for Healthcare Research and Quality (AHRQ) [8] from 2016 to 2018. The unweighted sample contains around seven million observations, while the weighted sample contains 35 million records for each year. Weights were provided by NIS to calculate national estimates. As the database contains a de-identified patient sample, it is deemed to be exempt from institutional board review.

Inclusion and exclusion criteria

All patients with age ≥18 years admitted to the hospital with a principal diagnosis of DKA were included in the study. DKA group was further divided into those with ESKD and those with no CKD/ESKD. We used the International Classification of Diseases, Tenth Revision, Clinical Modification (ICD-10 CM) codes to retrieve patient samples and ICD-10 procedure codes for mechanical ventilation. Patients with missing demographic data, age <18 years, and CKD stages 1 to 5 were excluded from the study. A detailed code summary is provided in Table 1.

**Table 1:** ICD-10 CM codes ICD-10 CM: International Classification of Diseases, Tenth Revision, Clinical Modification; CKD: Chronic kidney disease; DKA: Diabetic ketoacidosis; ESKD: End-stage kidney disease

Variable	ICD-10 CM code
DKA	E08.1X, E09.1X, E10.1X, E11.1X, E13.1X
CKD1	N18.1
CKD2	N18.2
CKD3	N18.3X
CKD4	N18.4
CKD5	N18.5
ESKD	N18.6
Mechanical Ventilation	5A1945Z, 5A1955Z, 5A1935Z, 0BH13EZ, 0BH17EZ, 0BH18EZ

Covariates

NIS data sample contains data regarding in-hospital outcomes, procedures, and other discharge-related information. Variables included (a) Patient characteristics: age, race, sex, comorbidities, disposition, (b) Hospital demographics: location, teaching status, bed size, region, and (c) Illness severity: length of stay (LOS), mortality, mechanical ventilation.

Study outcomes

The primary outcome was in-hospital mortality in patients with DKA and ESKD compared to DKA without CKD/ ESKD. Secondary outcomes were: (a) mechanical ventilation rates and (b) length of stay (LOS), hospitalization costs, and discharge disposition.

Statistical methods

All statistical analyses were conducted utilizing Stata Statistical Software: Release 17, 2021 (Stata Corp., College Station, Texas, US). Weights provided in the NIS dataset were applied for all analyses. All p-values were two-sided, and 0.05 was considered statistically significant. In both the groups, categorical variables were compared using the Chi-square test, while continuous variables (age, LOS, total charge) were compared using linear regression. Multivariate logistic regression model was used to adjust for potential confounders for mortality and mechanical ventilation while multivariate linear regression was used to adjust for confounders for LOS and hospitalization charges. 

## Results

Patient characteristics

**Figure 1:**
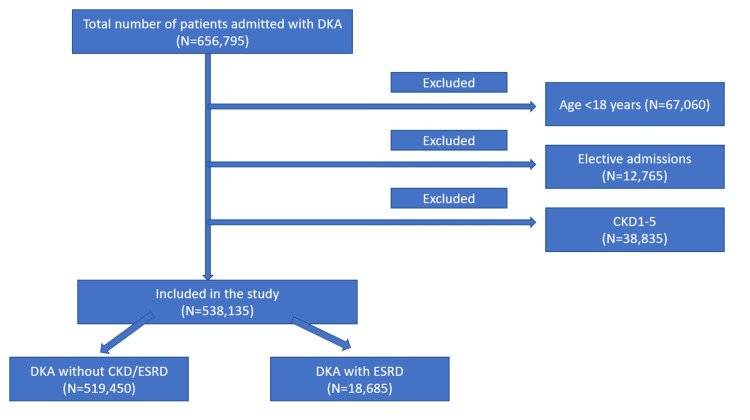
Inclusion and exclusion criteria DKA: Diabetic ketoacidosis; CKD: Chronic kidney disease; ESKD: End-stage kidney disease

A total of 538,135 patients were included in the study after excluding the patients as shown in Figure 1, of which 18,685 (3.74%) patients had DKA with ESKD. Age groups 30-49 years and 50-69 years had higher prevalence of ESKD (52.74% vs 36.56%) and (31.58% vs 24.2%) in contrast to age <30 (11.856% vs 33.9%) years and age >70 (4.12% vs 5.34%) years. Females comprised 52.4% of the total population. The mean age was 43.7 years (SD 13.6) and 40.4 years (SD 17.2) in the ESKD group and preserved kidney function group (p<0.001). ESKD group had a higher number of African American population (41.26% vs. 25.94%, p<0.001).

A large number of patients in the ESKD group had Medicare insurance (63.2% vs. 20.24%), while the no CKD/ESKD group had a higher number of Medicaid insurance (36.38% vs. 24.29%), private insurance (29.36% vs. 11.16%), and self-pay (13.83% vs. 1.35%) with p<0.001. The rest of the patient-level characteristics are shown in Table 2.

**Table 2:** Patient characteristics CKD: Chronic kidney disease; ESKD: End-stage kidney disease

Variable	DKA with no CKD/ESKD	DKA with ESKD	p-value
Sample (N=538,135)	519,450(96.53%)	18,685(3.47%)	
Age (years)			<0.001
18-29	(176,094)33.9%	(2,160)11.56%	
30-49	(189,911)36.56%	(9,854)52.74%	
50-69	(125,707)24.2%	(5,901)31.58%	
≥70	(27,738)5.34%	(770)4.12%	
Mean Age (SD)			<0.001
Female	40.4 (17.2)	43.7 (13.6)	
Male	40.01 (15.6)	46.2 (12.7)	
Sex (Female)	(254,375)48.97%	(9,791)52.4%	<0.001
Race			<0.001
Caucasian	(296,762)57.13%	(7,993)42.78%	
African American	(134,745)25.94%	(7,773)41.26%	
Hispanic	(64,152)12.35%	(2,422)12.69%	
Asian or Pacific Islander	(5,818)1.12%	(224)1.2%	
Native American	(5,298)1.02%	(121)0.65%	
Other	(12,675)2.44%	(265)1.42%	
Income in patients zip code			<0.001
0-25^th^ percentile	(200,767)38.65%	(8,079)43.24%	
26-50^th^ percentile	(142,953)27.52%	(5,028)26.91%	
51-75^th^ percentile	(109,084)21%	(3,546)18.98%	
>76^th^ percentile	(66,697)12.84%	(2,029)10.86%	
Insurance			<0.001
Medicare	(106,072)20.42%	(11,809)63.2%	
Medicaid	(188,976)36.38%	(4,538)24.29%	
Private	(152,511)29.36%	(2,086)11.16%	
Self-pay	(71,841)13.83%	(254)1.35%	
Hospital Region			<0.001
Northeast	(76,047)14.64%	(3,100)16.59%	
Midwest	(111,058)21.38%	(4,320)23.12%	
South	(229,233)44.13%	(8,159)43.67%	
West	(103,111)19.85%	(3,105)16.62%	
Teaching status and location			<0.001
Rural	(62,282)11.99%	(1,130)6.05%	
Urban non-teaching	(130,798)25.18%	(4,034)21.59%	
Urban teaching	(326,370)62.83%	(13,520)72.36%	
Hospital bed size			<0.001
Small	(115,682)22.27%	(2,906)17.55%	
Medium	(158,952)30.6%	(5,450)29.17%	
Large	(244,817)47.13%	(9,955)53.28%	

In-hospital outcomes

While ESKD group had higher mortality (0.83% vs 0.38%) and mechanical ventilation (4.28% vs 1.74%), adjusted odds ratio for mortality 1.12 (95%CI 0.75-1.68), p=0.56, and mechanical ventilation 1.11 (95%CI 0.92-1.33), p=0.25, were not statistically significant. These results are represented in Table 3. 

**Table 3:** In-hospital outcomes CKD: Chronic kidney disease; ESKD: End-stage kidney disease; SNF: Skilled nursing facility; AMA: Discharge against medical advice ^1^Adjusted for age, sex, race, Elixhauser comorbidity score, insurance status, hospital location, and characteristics

Variable	DKA with no CKD/ESKD	DKA with ESKD	p-Value
Disposition			<0.001
Regular	(431,144) 83%	(12,345)66.07%	
SNF	(24,674) 4.75%	(2,459)13.16%	
Home health	(30,959) 5.96%	(2,812)15.05%	
AMA	(32,622) 6.28%	(1,069)5.72%	
In-hospital mortality	(1,974) 0.38%	(155)0.83%	<0.001
	Adjusted odds ratio^1^1.12 (CI 0.75-1.68)		0.56
Mechanical ventilation	(9,038) 1.74%	(800)4.28%	<0.001
	Adjusted odds ratio^1^ 1.11 (CI 0.92-1.33)		0.25
Mean total hospitalization charge ($)	$30,196	$56,845	<0.001
	Adjusted total charge^1^ $14,882.2 higher		<0.001
Mean length of stay (days)	3.12	5.36	<0.001
	Adjusted length of stay^1^ 0.87 day higher		<0.001

The mean LOS and mean hospitalization charge in the ESKD group were 5.36 days and $56,845, respectively, while in the group with no CKD/ ESKD, these were 3.12 days and $30,196, respectively. After adjusting to potential confounders as described in the methods section, the ESKD group had 0.87 days longer LOS and incurred $14,882.2 higher hospitalization costs as compared to the group with no CKD/ ESKD.

The ESKD group required higher rates of nursing home transfer (13.16% vs. 4.75%) and home health (15.05% vs. 5.96%) at discharge with p<0.001 when compared to patients with no CKD/ ESKD.

## Discussion

We analyzed over 106.9 million patients admitted between 2016 to 2018 utilizing the largest publicly available national database. Over 519,450 patients were admitted for DKA, of which only 3.47% (18,685) patients had a concurrent diagnosis of ESKD. To our knowledge, this is the largest study to date to report in-hospital outcomes in patients with DKA and ESKD.

In our study, we found that a relatively low number of patients, 3.47% (18,685/538,135), had DKA with ESKD. The primary pathophysiology of DKA is insulin deficiency. In patients with renal failure, insulin clearance is delayed [9]; this can protect against developing DKA in ESKD. On the other hand, the diagnosis can be missed due to the correction of hyperglycemia and acidosis in ESKD with hemodialysis [10].

We found a higher incidence of DKA in females in the ESKD group (52.4% vs. 48.97%) than in the patients with no CKD/ ESKD. Other studies supported this finding where DKA with ESKD was more prevalent in females [6]. Several international studies have shown that the female sex is an independent risk factor for developing DKA regardless of ESKD [11-14]. Intentional skipping of insulin doses due to fear of gaining weight, higher prevalence of eating, and psychiatric disorders may lead to poor disease control, which could explain the higher incidence and younger age in the female subset [11,15,16]. The younger population, i.e., age 18-29 years, formed a major portion of the DKA patients with no CKD/ESRD (33.9% vs. 11.56%) than in the DKA with ESKD. Whereas the DKA with ESKD was mainly constituted by a relatively older population (52.74% in 30-49 years, 31.58% in 50-69 years). In their study, Galindo et al. found similar discrepancies in ages, with the mean age being 54 years in the ESKD group vs. 35 years in the group with no CKD/ESKD [6]. DKA is more common in type 1 diabetes mellitus [17], and ESKD develops a couple of years to decades after poorly-controlled diabetes, which explains the age difference between the two groups [18].

Our study showed that the incidence of DKA in ESKD was similar in the African American and Caucasian populations similar to the study done by Galindo et al [6]; however, we did find that African American patients with DKA have a higher prevalence of ESKD (41.26% vs. 25.94%) than the group with no CKD/ESKD. Studies done by Norton et al. and Delamater et al. showed that the African American population has 3.5 times higher risk of progression from early CKD to ESKD. They have a higher acute/chronic complications rate than the Caucasian population [19,20]. 

A higher percentage of the DKA with ESKD group were patients with lower income (43.24%). This emphasizes the financial impact of dialysis and diabetes on patients [21]. ESKD patients are eligible for Medicare regardless of age, which explains the higher percentage of Medicare insurance (63.2%) in the ESKD group.

Our study found no significant difference in-hospital mortality and mechanical ventilation between the ESKD group and the group with no CKD/ESKD. The no difference in in-hospital mortality is consistent with a single-center study done by Galindo et al., which found higher 30-day mortality and one-year mortality in the ESKD group [6]. We found that patients with no CKD/ ESRD were more likely to be discharged home (83% vs. 66.07%) than ESKD patients. The DKA with ESKD patients were more likely to be discharged to a skilled nursing facility (13.16%) or with home health (15.05%). This emphasizes the impact of DKA on short-term morbidity and long-term morbidity and mortality. However, in contrast to our study, Galindo et al. also reported increased mechanical ventilation in the ESKD group; this was explained by volume overload and pulmonary edema. It's worth noting an increased trend toward increased mechanical ventilation in the ESKD group (1.74% vs 4.28%) in our study as well. This emphasizes the need for dedicated DKA management protocols in ESKD groups.

We also found that DKA patients in hospitals located in the Northeast and Midwest regions of the US had higher underlying ESKD whereas, in the southern and eastern parts of the US, DKA with preserved kidney function was higher. This may be due to patient characteristics like agew, gender, race, and socioeconomic status in these regions. Larger metropolitan areas with larger hospitals had a higher prevalence of DKA cases with underlying ESKD, probably due to more complex cases being referred to larger centers for treatment.

Implications on healthcare

ESKD patients are at increased risk of hypoglycemic events and hypervolemia [6] and require dialysis, leading to a longer LOS and increasing the cost of hospitalization. The adjusted mean total hospitalization charge in patients with DKA and ESKD was $14,882 higher than in patients with no CKD/ ESKD. Davis et al., in their study, showed that CKD patients have higher adverse glucose events when treating DKA patients [9]. Hurtardo et al. found that ESKD is an independent risk factor for readmission in DKA patients [22]. Galindo et al., in their study, also found that the hospitalization costs were significantly higher in the ESKD cohort (median $50,844 vs. median $14,252, p<0.001) [6]. Similarly, the adjusted mean length of hospital stay in the ESKD group was 0.87 days higher than patients with preserved kidney function.

Limitations

As with any cross-sectional study, we cannot establish causality but only associations. Our study is based on a database based on ICD-10 coding, which can include possible coding errors/risk factors or diagnoses not entered into the database. Given that CKD stage 1 or 2 is very commonly missed in coding or diagnosis, this limitation should be considered while interpreting the study. We could not evaluate social barriers to discharge, outpatient resource accessibility, and medication compliance. NIS does not record labs and imaging data. Data on acute kidney onjury and specific electrolyte and glucose abnormalities were not evaluated, which are potential confounders in our study.

## Conclusions

Our study showed that only 3.47% of patients admitted with DKA has underlying ESKD. Women and African American patients were significantly higher in ESKD group and 33.93% had non-routine discharge. While ESKD group had higher length of stay and total hospitalization cost, there was no difference in in-hospital mortality and mechanical ventilation. There is a dearth of evidence-based guidance regarding DKA management in CKD and ESRD. Further studies looking into measures in the management of DKA in ESRD will help develop guidelines in management, decreasing morbidity and cost of hospitalization.
